# Novel findings in context of molecular diversity and abundance of bacteriophages in wastewater environments of Riyadh, Saudi Arabia

**DOI:** 10.1371/journal.pone.0273343

**Published:** 2022-08-18

**Authors:** Fahad Alanazi, Islam Nour, Atif Hanif, Ibrahim Al-Ashkar, Reem M. Aljowaie, Saleh Eifan

**Affiliations:** 1 Botany and Microbiology Department, College of Science, King Saud University, Riyadh, Saudi Arabia; 2 Department of Plant Production, College of Food and Agriculture Sciences, King Saud University, Riyadh, Saudi Arabia; Friedrich Schiller University, GERMANY

## Abstract

The diversity among bacteriophages depends on different factors like ecology, temperature conditions and genetic pool. Current study focused on isolation, identification and diversity of phages from 34 sewage water samples collected from two different wastewater treatment plants (WWTPs), King Saud University wastewater treatment plants (KSU-WWTP) and Manfoha wastewater treatment plants (MN-WWTP) in Riyadh, Saudi Arabia. Samples were analyzed by PCR and Next Generation Sequencing (NGS). *Siphoviridae*, *Podoviridae* and *Myoviridae* families were detected by family-specific PCR and highest prevalence of *Myoviridae* 29.40% was found at MN-WWTP followed by 11.76% at KSU-WWTP. *Siphoviridae* was detected 11.76% at MN-WWTP and 5.88% at KSU-WWTP. Lowest prevalence for *Podoviridae* family (5.88%) was recorded at MN-WWTP. Significant influence of temporal variations on prevalence of *Myoviridae* and *Siphoviridae* was detected in both WWTP and MN-WWTP, respectively. Highest phage prevalence was obtained in August (75%), followed by September (50%). Highest phage prevalence was recorded at a temperature range of 29–33°C. Significant influence of temperature on the prevalence of *Myoviridae* phages was detected at MN-WWTP. Four bacteriophages with various abundance levels were identified by NGS. Cronobacter virus Esp2949-1 was found first time with highest abundance (4.41%) in wastewater of Riyadh. Bordetella virus BPP1 (4.14%), Dickeya virus Limestone (1.55%) and Ralstonia virus RSA1 (1.04%) were also detected from samples of MN-WWTP. Highest occurrence of Bordetella virus BPP1 (67%) and (33.33%) was recorded at KSU-WWTP and MN-WWTP, respectively. Highest Bordetella virus BPP1 occurrence was recorded in September (50%) followed by August (40%). The findings of study showed new insights of phage diversity from wastewater sources and further large-scale data studies are suggested for comprehensive understanding.

## 1. Introduction

Bacteriophages were identified with a potential capacity and specificity to infect certain bacteria in various environments [1, 2]. Bacteriophages are known as the most widely distributed population with 10^31^ phages and over a hundred million species frequently found in contaminated waters such as sewage water [3]. Different varieties of phages were reported in ecosystems of various locations and found related to bacterial host diversity [4, 5]. For instance, 8.52 genetically different *Escherichia coli* per 10 *E*. *coli* isolates showed the great rate of *E*. *coli* diversity that mainly led to great phage diversity [6]. Moreover, bacteriophages play a key role in bacterial evolution by phage infection [7].

DNA amplification and sequencing have been used as a reliable and sensitive tool for molecular characterization of bacteriophages [8]. Over a 95% of bacteriophages considered to carry double-stranded DNA as their genetic material and infect over 130 bacterial genera [8]. Bacteriophages with enormous genome distribution among their hosts have the ability to evolve along with their host cell evolution. Furthermore, phages genome variations studies could help us to fully understand the evolutionary stages [9]. Whole genome sequencing is considered as a reliable tool to identify bacteriophages [10]. However, next generation sequencing (NGS) is currently known as the most accurate genome sequencing method for sequences determination and provides data that can be compared with published genetic databases. This data comparison could be utilized to detect mutations, genetic variations and evolutionary stages by phylogenetic analysis [11].

On the other hand, temperature variations could affect the phage diversity in different habitats [12, 13]. Moreover, other environmental factors could lead to dependent and independent biological changes resulting in microbial diversity [14–16]. Furthermore, population density is another significant factor that affects microbial diversity and consequently phages population [17]. Impact of environmental changes on phage diversity and abundance has been reported during wastewater treatment processes [18]. Therefore, the current study aimed at detection of bacteriophage families from wastewater samples by using family-specific PCR analysis and deep molecular characterization of bacteriophage isolates by means of metagenomics approach for the assessment of phage diversity.

## 2. Materials and methods

### 2.1. Water sampling

A total of 34 untreated wastewater samples were collected with a frequency of one sample per week from KSU-WWTP (24°43’33.8"N 46°36’27.9"E) and MN-WWTP (24°35’12.0"N 46°43’53.0"E) in sterilized 500 ml safety containers during August—November, 2020 (i.e. seventeen samples from each WWTP). Temperatures were recorded on sample collection days from the accuweather (https://www.accuweather.com/en/sa/riyadh) for Riyadh region, Saudi Arabia.

### 2.2. Concentration of bacteriophages

Bacteriophages were concentrated by PEG precipitation method [19]. Briefly, 200ml of sample was mixed with 25ml of glycine buffer, centrifuged at 8000 *×g* for 30 minutes and supernatant was filtrated by using 0.22-μm syringe filter. PEG 80g/L and NaCl 17.5g/L was added to the 200ml filtrate, stirred at 100 *×g* at room temperature for 10 hours and centrifuged at 13,000 *×g* for 30 minutes. The pellet was dissolved in 1ml of phosphate buffer saline (PBS) and stored at -80°C.

### 2.3. Bacteriophages nucleic acid extraction, PCR amplification and electrophoresis

Phage DNA was extracted by PowerViral^®^ Environmental RNA/DNA Isolation Kit (MO BIO, Carlsbad, CA, USA), following the manufacturer’s instructions. A total of 50-μL PCR mixture including 25 μL 1X Phusion Master Mix (Thermo Fisher Scientific, USA), 200-nM forward primer, 200-nM reverse primer and 5 μL template DNA was prepared. PCR was performed with initial denaturation at 98 °C for 30 s, followed by 40 cycles of 98 °C for 10 s, Tm °C (Table 1) for 30 s and 72°C for 30 s, and final extension at 72 °C for 10 min. Amplicons were resolved on 2% agarose gel and results were analyzed. The procedure of viral recovery efficiency from water samples was established in our laboratory with a detection limit of 10 copy per single run [19].

**Table 1:** PCR primers used in phage isolation.

Gene	family	Amplicon size (bp)	Tm (°C)	Sequence	Reference
G23 major capsid protein	Myoviridae	500	54	CTF-F: GAYHTIKSIGGIGTICARCCIATG	[20]
				CTF-R: GCIYKIARRTCYTGIGCIARYTC	
DNA Polymerase		476	50	DGF-F: GCWGGTGCWTATGTHAARGAACC	[21]
				DGF-R: CCWGASARAGTAATKGCYTCWGC	
Major coat protein		1278	56.3	MCF-1F: CTGGTCGTGTTCAGCAGACT	[22]
				MCF-1R AGCCATAAGAGCAGGATCGC	
Major coat protein	Siphoviridae	459	56.4	MCF-2F: GCGTGATGGTTGGGATGGTA	
				MCF-2R: GACGCTCAATCTGACGACCA	
	Podoviridae	774	56.4	MCF-3F: CCGCGATTGCGAGCATTAAA	
				MCF-3R: CGGTCTGAATGTTCACCGGA	

### 2.4. Library preparation and sequencing

Illumina Nextera XT library preparation kit was used to prepare DNA libraries. DNA Libraries was quantified by Invitrogen Qubit assay (Thermo Fisher Scientific, USA). DNA libraries were sequenced by using Illumina HiSeq platform targeting 4 million read pairs (pair-ended 2 x 150bp) per sample.

### 2.5. NGS sequence analysis

Unassembled sequencing reads were directly analyzed by bioinformatics platform as described by [23–25] for multi-kingdom microbiome analysis and quantification of organisms’ relative abundance. The system utilizes curated genome databases and a high-performance data-mining algorithm that rapidly disambiguates hundreds of millions of metagenomics sequence reads into the discrete microorganisms.

### 2.6. Prevalence of bacteriophage families and species in WWTPs

The prevalence of bacteriophage families in water samples was determined by PCR and calculated according to the following formula: ${Prev}_{f}=\frac{C_{p}}{T}*100$, where *Prev*_*f*_ is the prevalence of bacteriophage family, *C*_*p*_ is the count of PCR positive samples per family and *T* is the total number of tested samples. Relative abundance of NGS detected phages was calculated according to the following formula; number of phages-specific sequences divided by the total number of obtained sequences by NGS (for PCR-positive samples).

### 2.7. Statistical analysis

The variables, including family level, temporal level (August—November), water source (KSU-WWTP and MN-WWTP), and meteorological condition (include the temperature ranges: 18–22 °C, >22–26 °C, >26–29 °C, >29–33°c and >33), were analyzed. One-way analysis of variance was performed to test the significant impact of temperature ranges and temporal variations on detected phage families and prevalence of both WWTPs. The relationships between both sampling locations (dependent variables), and temperatures and temporal variation (independent variables) were fitted using linear curve fitting. Pearson’s correlation coefficient matrix was conducted to assess the potential relationships between the phage positive samples detected by PCR and phages sequence abundance obtained by NGS. All the statistical analyses were performed by using the XL-STAT statistical package software (Ver. 2019, Excel Add-ins soft SARL, New York, NY, USA).

## 3. Results

### 3.1. PCR-based detection of bacteriophages in WWTPs

**Fig 1:**
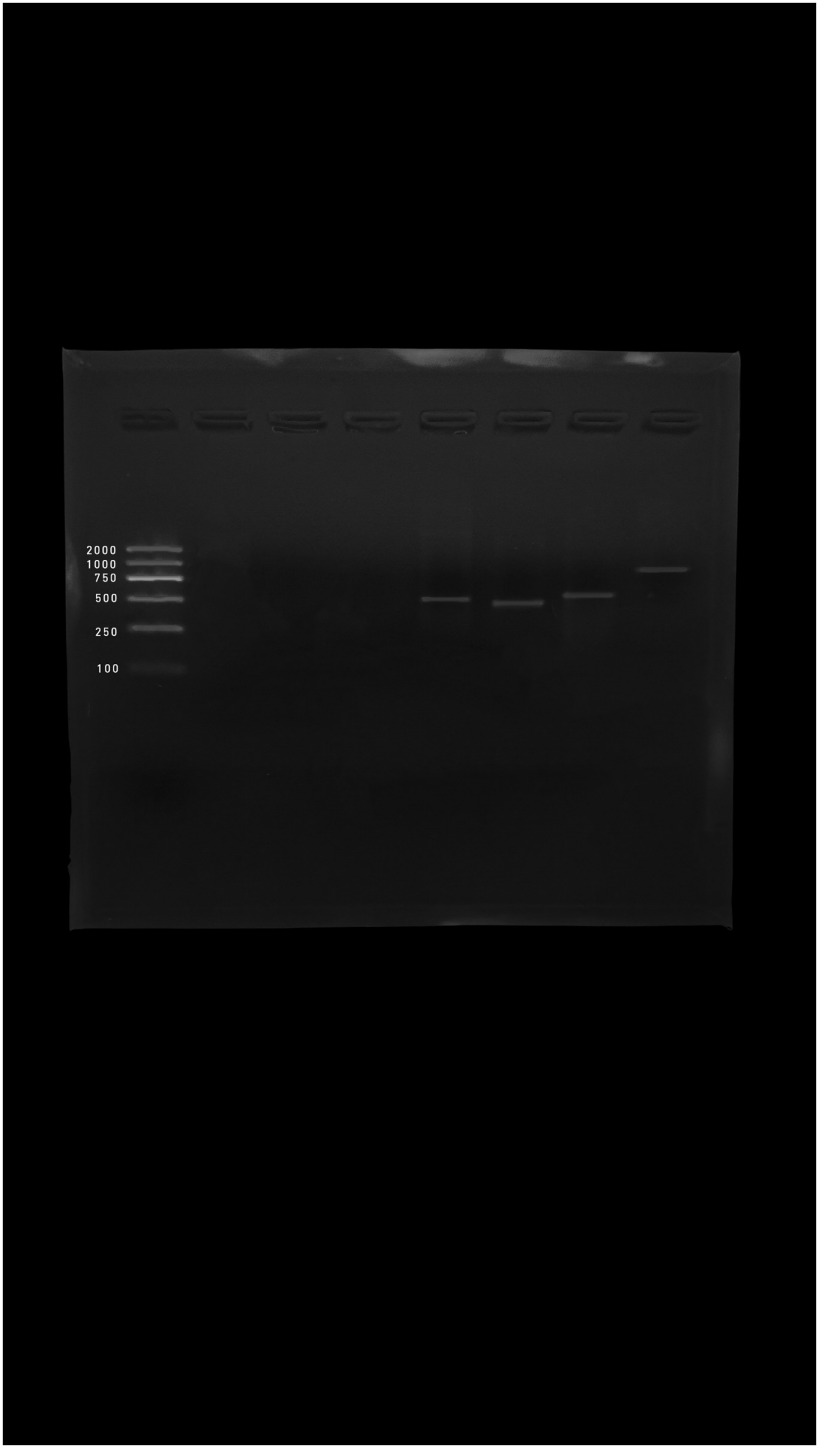
PCR product of raw water sample*s* from MN-WWTP. (A) Lane 1: DNA ladder (100–2000 bp), Lane 2: negative control, Lane 3,4: negative samples, Lane 5: 476 bp (*Myoviridae*, DGF primer), Lane 6: 459 bp (*Siphoviridae*, MCF-2 primer), Lane 7: 500 bp (*Myoviridae*, CTF primer) and Lane 8: 774 bp (*Podoviridae*, MCF-3 primer).

**Fig 2:**
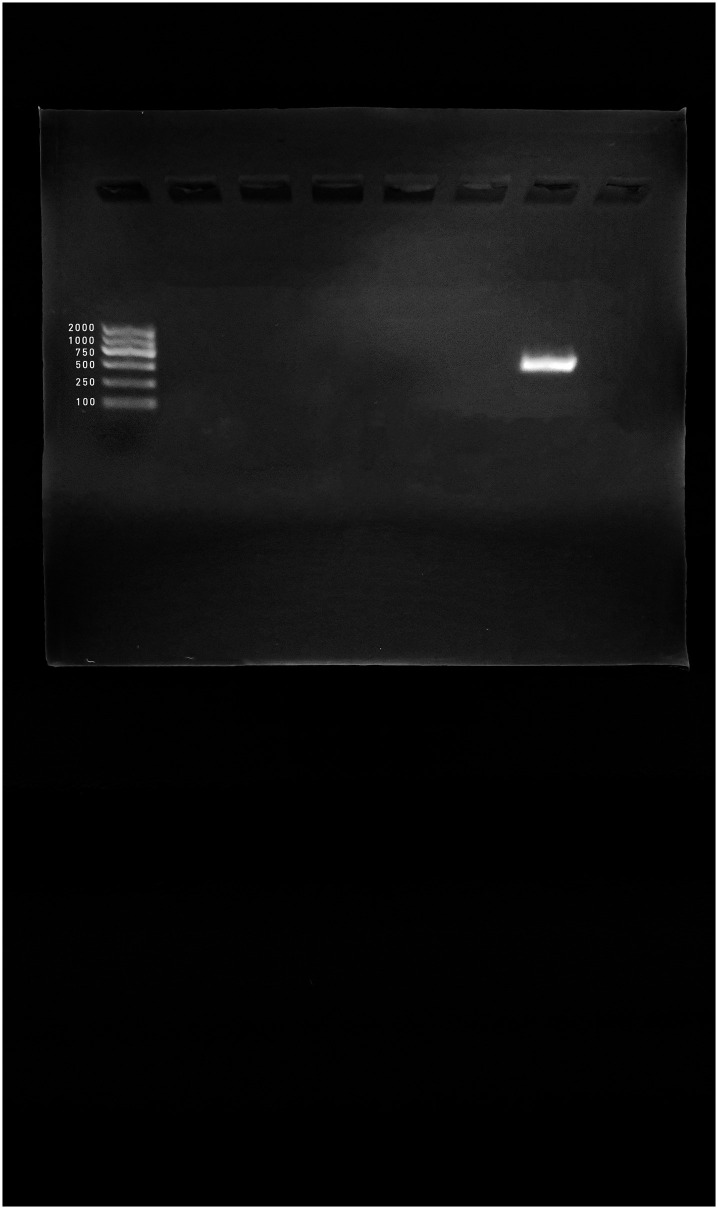
PCR product of raw water sample*s* from KSU-WWTP. Lane 1: DNA ladder, Lane 2: negative control, Lane 3–6 and 8: negative samples, Lane 7: 500 bp (*Myoviridae*, CTF primer).

*Siphoviridae* and *Podoviridae* families were detected with amplicon sizes 459 bp and 774 bp, respectively (Fig 1). Moreover, *Myoviridae* was detected with different amplicons sizes 476 bp (Fig 1A), 500 bp (Figs 1 and 2, S1A, S1B, S1D and S1E and S2B Figs), and 1278 bp (S1D Fig).

### 3.2. Prevalence of phage families in WWTPs

**Fig 3:**
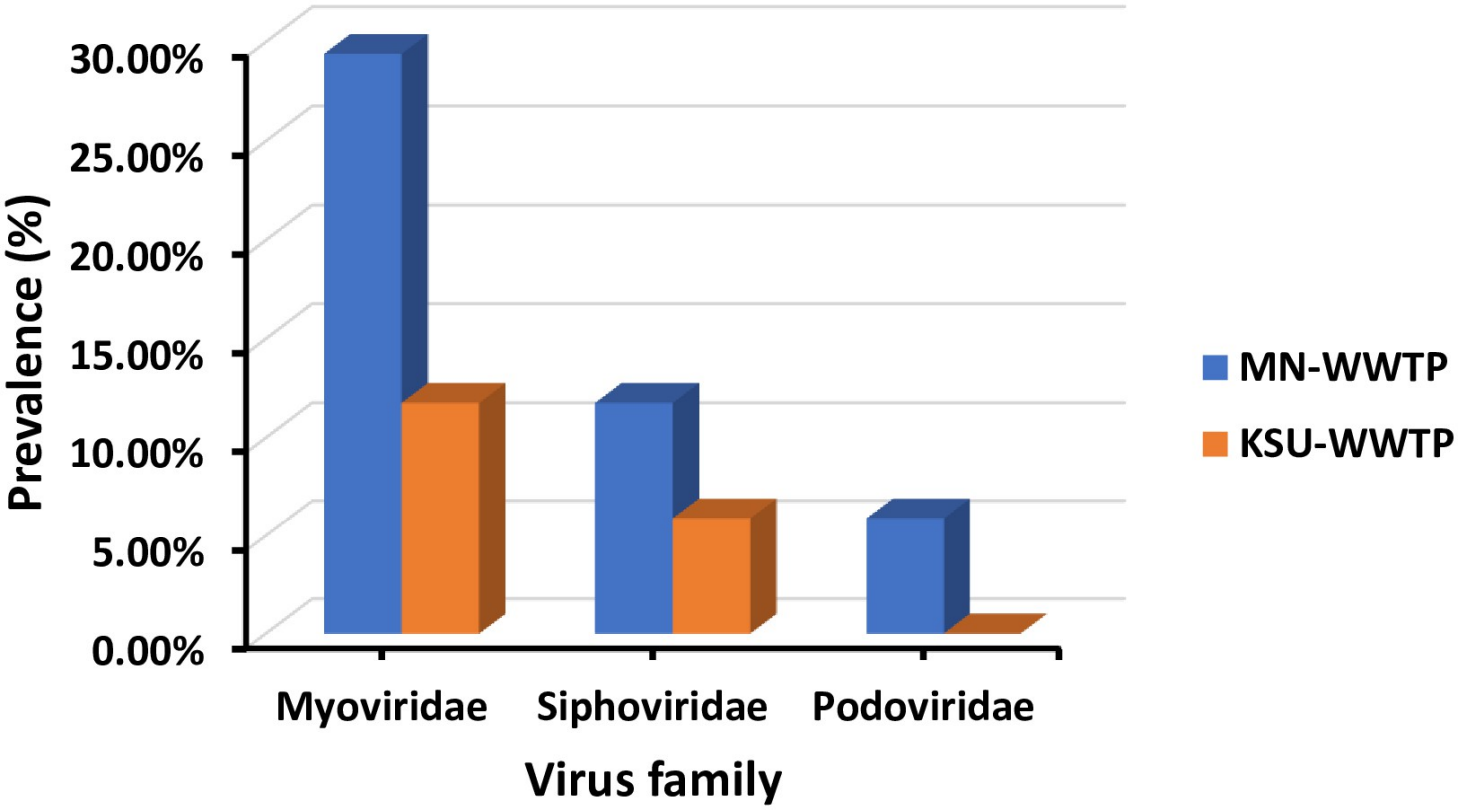
Prevalence of phage families in WWTPs.

Bacteriophages’ prevalence results varied according to water source and detected phage families. MN-WWTP depicted the highest prevalence of all phage families. Moreover, *Myoviridae* was recorded with highest prevalence of 29.40% among all families in MN-WWTP in comparison to KSU-WWTP (11.76%). *Siphoviridae* prevalence recorded 11.76% in MN-WWTP and 5.88% in KSU-WWTP whereas *Podoviridae* depicted lowest prevalence (5.88%) in MN-WWTP and no prevalence was recorded at KSU-WWTP (Fig 3).

### 3.3. Temporal variations influence on bacteriophage prevalence

**Fig 4:**
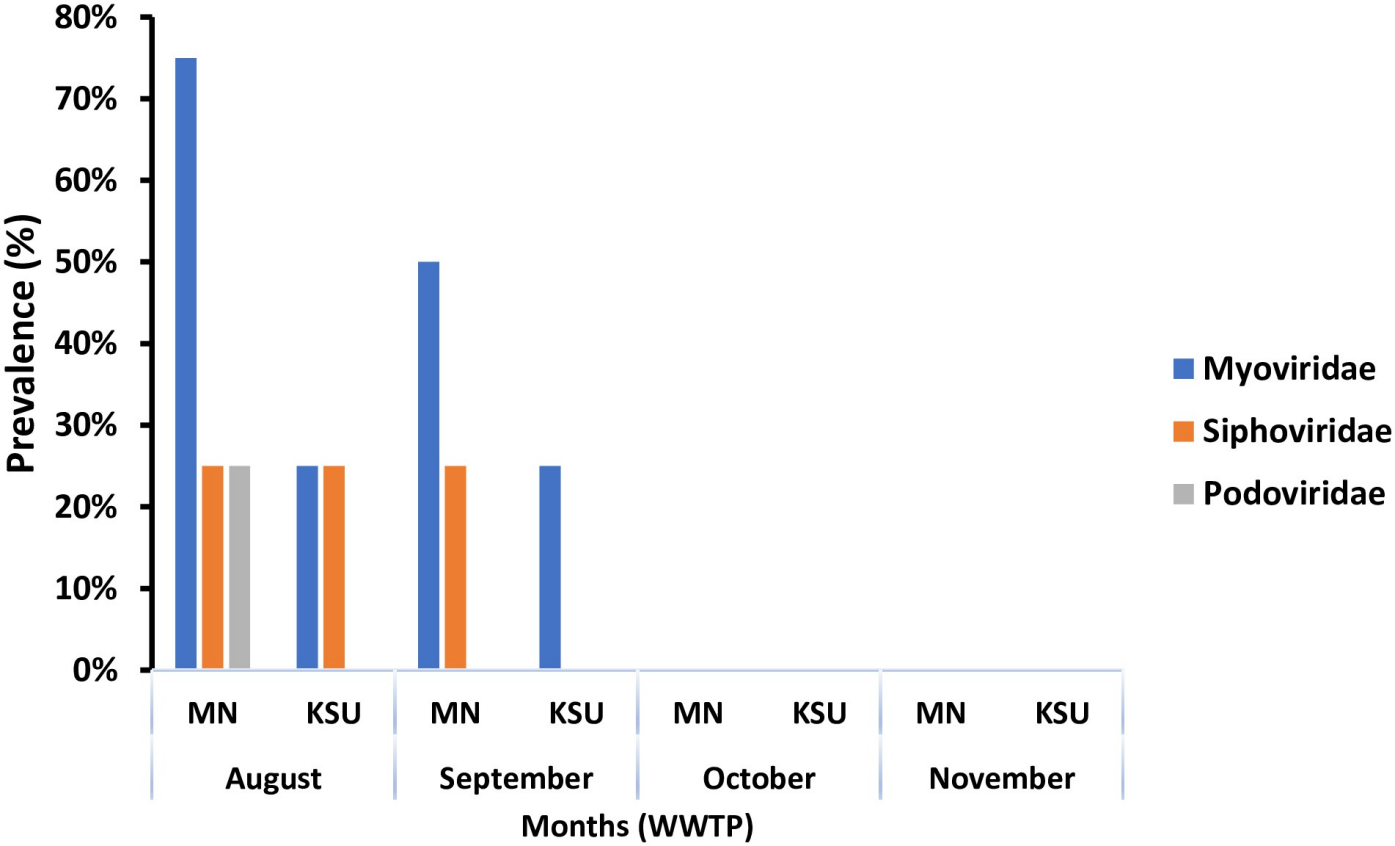
Temporal prevalence of phage families in WWTPs during four-month period.

Phage prevalence was affected temporally that showed the highest phage prevalence in August (75%), followed by September (50%). However, no bacteriophages were found during months of October and November. *Myoviridae* was detected with highest frequency (75%), whereas *Siphoviridae* and *Podoviridae* were recorded with lowest frequencies (25%) in August. Furthermore, higher phages prevalence recovered in August in comparison to month of September from MN-WWTP. *Podoviridae* was detected only in August with 25% prevalence in MN-WWTP (Fig 4). Significant influence of temporal variations on prevalence of *Myoviridae* and *Siphoviridae* was found in both WWTPs and MN-WWTP, respectively (Table 2).

**Table 2:** The significance of the influence of temperature on prevalence of phage families in wastewater treatment plants.

Phage family	Sampling Area	R^2^	RMSE^Ϯ^	Equation
*Myoviridae*	MN-WWTP	**0.896** *	0.592	MN-Myo = 4–1.1*M^‡^
	KSU-WWTP	**0.8** *	0.316	KSU-Myo = 1.5–0.4*M
*Siphoviridae*	MN-WWTP	**0.8** *	0.316	MN-Sipho = 1.5–0.4*M
	KSU-WWTP	0.6	0.387	KSU-Sipho = 1–0.3*M
*Podoviridae*	MN-WWTP	0.6	0.387	MN-Podo = 1–0.3*M
	KSU-WWTP	--	--	--

MN-Myo: refers to *Myoviridae* phages detected in MN-WWTP,

^Ϯ^: RMSE denotes the root mean squared error, that is an absolute measure of fit.

^‡^: M denotes month,

*: significant correlation.

### 3.4. Temperature variations impact on prevalence of PCR detected phage families

**Fig 5:**
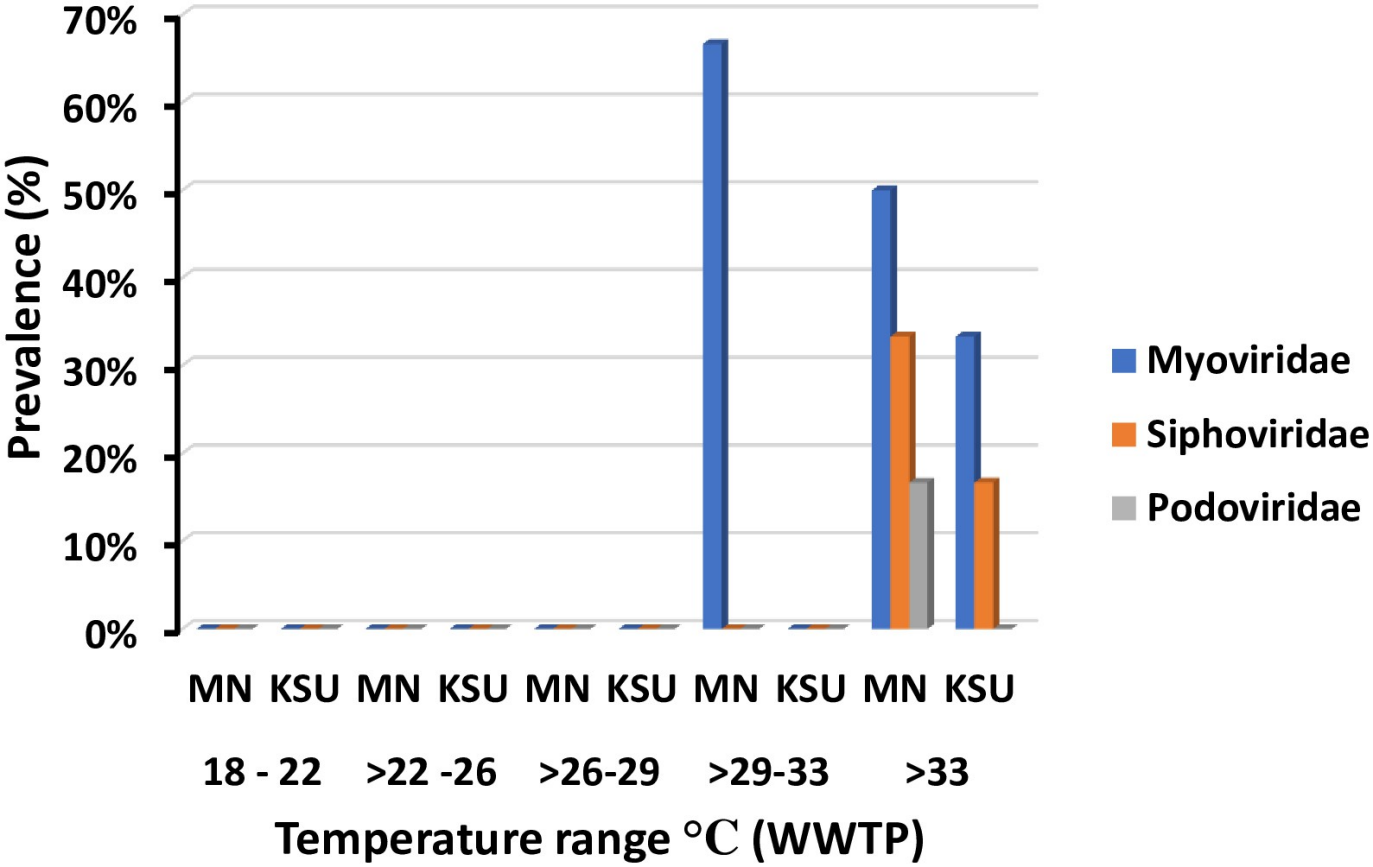
Frequency of phage families across different temperature ranges in WWTP.

The highest prevalence of phage families was found at the temperature range >29–33°C. However, phages were undetectable at ≤29°C. Moreover, *Myoviridae* showed highest prevalence at >29–33°C in MN-WWTP (67%). *Siphoviridae* and *Podoviridae* displayed highest prevalence of 33% and 17%, respectively at >33°C. Greater frequency of *Myoviridae* and *Siphoviridae* was observed in MN-WWTP in comparison to KSU-WWTP at >33°C (Fig 5). Significant influence of temperature on prevalence of *Myoviridae* family was detected in MN-WWTP (Table 3).

**Table 3:** The significance of the influence of temperature on prevalence of phage families in wastewater treatment plants.

Phage family	sampling Area	R^2^	RMSE	Equation
*Myoviridae*	MN-WWTP	**0.8** *	0.73	MN-Myo = -5+0.2*T^‡^
	KSU-WWTP	0.5	0.73	KSU-Myo = -2.6+0.1*T
*Siphoviridae*	MN-WWTP	0.5	0.73	MN-Sipho = -2.6+0.1*T
	KSU-WWTP	0.5	0.365	KSU-Sipho = -1.3+0.05*T
*Podoviridae*	MN-WWTP	0.5	0.365	MN-Podo = -1.3+0.05*T
	KSU-WWTP	--	--	--

MN-Myo: refers to *Myoviridae* phages detected in MN-WWTP,

^‡^: T denotes temperature,

*: significant correlation.

### 3.5. Phage species relative abundance results

#### 3.5.1. NGS-based sequence relative abundance in PCR-based positive samples

**Fig 6:**
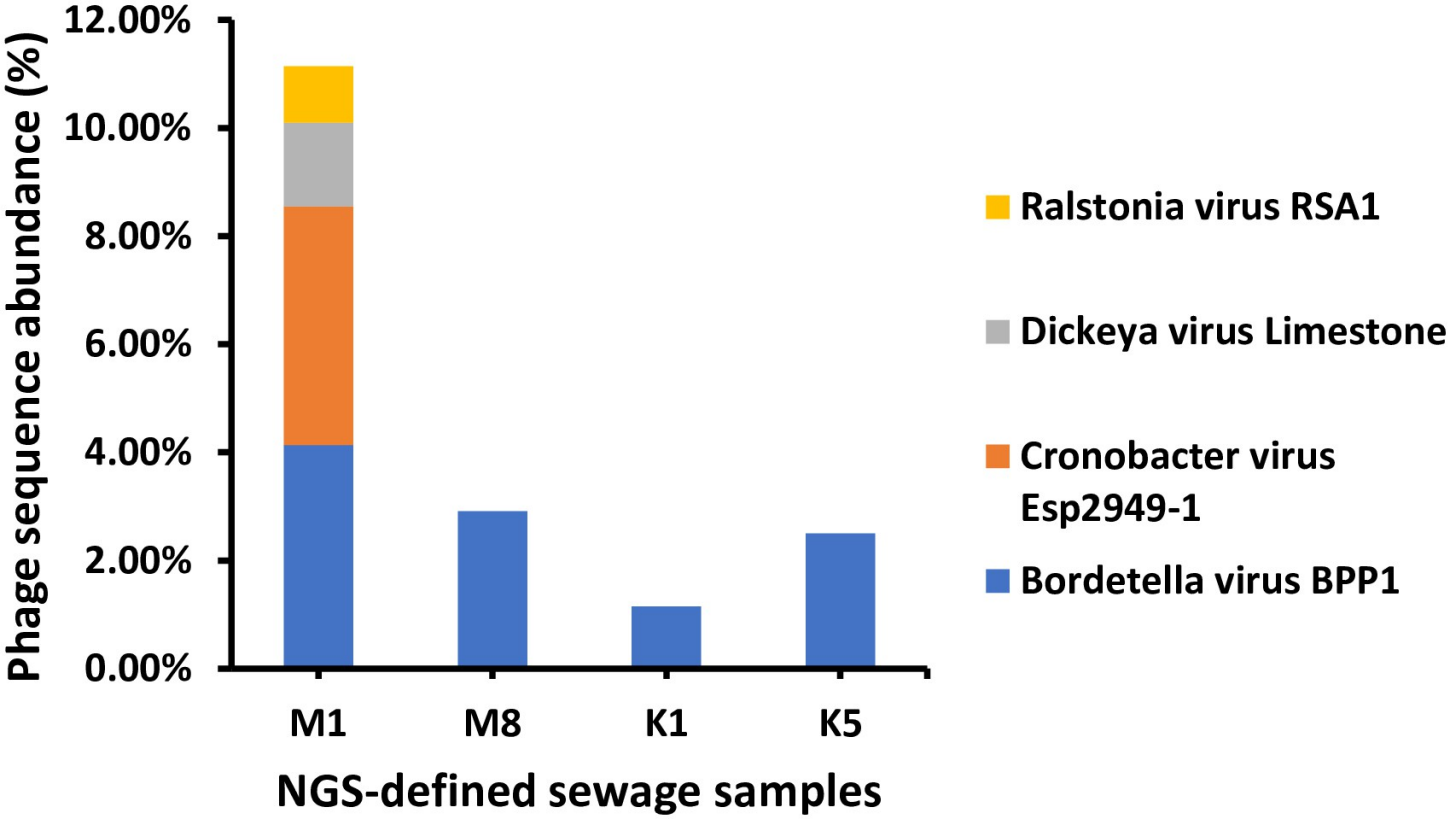
Relative abundance of phage sequence in NGS-defined sewage samples. M1: first sample from MN-WWTP, M8: eighth sample obtained from MN-WWTP, K1: first sample obtained from KSU-WWTP and K5: fifth sample obtained from KSU-WWTP.

Four bacteriophages were found with various abundances by NGS-Sequencing. Cronobacter virus Esp2949-1 was detected with the highest abundance (4.41%) only in M1 sample collected from MN-WWTP (Fig 6). Ralstonia virus RSA1 displayed the lowest sequence abundance of 1.04% in MN-WWTP raw water (Fig 6). Moreover, highest correlation was observed between K1 and K5 samples collected from KSU-WWTP. Bordetella virus BPP1 sequence abundance showed the highest correlation to KSU-WWTP samples (K1, *p* = 0.004 and K5, *p* = 0.007) and M8 sample collected from MN-WWTP (*p* = 0.012, Table 4).

**Table 4:** Pearson’s correlation matrix of different phage species in positive samples.

	**CV-Esp**	**DVL**	**RV-RSA1**	**M1**	**M8**	**K1**	**K5**
**BV-BPP1**	-0.333	-0.333	-0.333	0.502	**0.988**	**0.996**	**0.993**
**CV-Esp**	-	-0.333	-0.333	0.602	-0.329	-0.332	-0.331
**DVL**		-	-0.333	-0.458	-0.329	-0.332	-0.331
**RV-RSA1**			-	-0.647	-0.329	-0.332	-0.331
**M1**				-	0.507	0.506	0.507
**M8**					-	0.998	**0.999**
**K1**						-	**1.000**

BV-BPP1: Bordetella virus BPP1, CV-Esp: Cronobacter virus Esp2949-1, DVL: Dickeya virus Limestone, RV-RSA1: Ralstonia virus RSA1. Significant correlation values are displayed as bold numbers. Pearson’s correlation was found highest among positive samples from KSU-WWTP, while Bordetella virus BPP1 sequence abundance showed the highest correlation to samples from KSU-WWTP and M8.

#### 3.5.2. Occurrence of phage species in WWTPs

**Fig 7:**
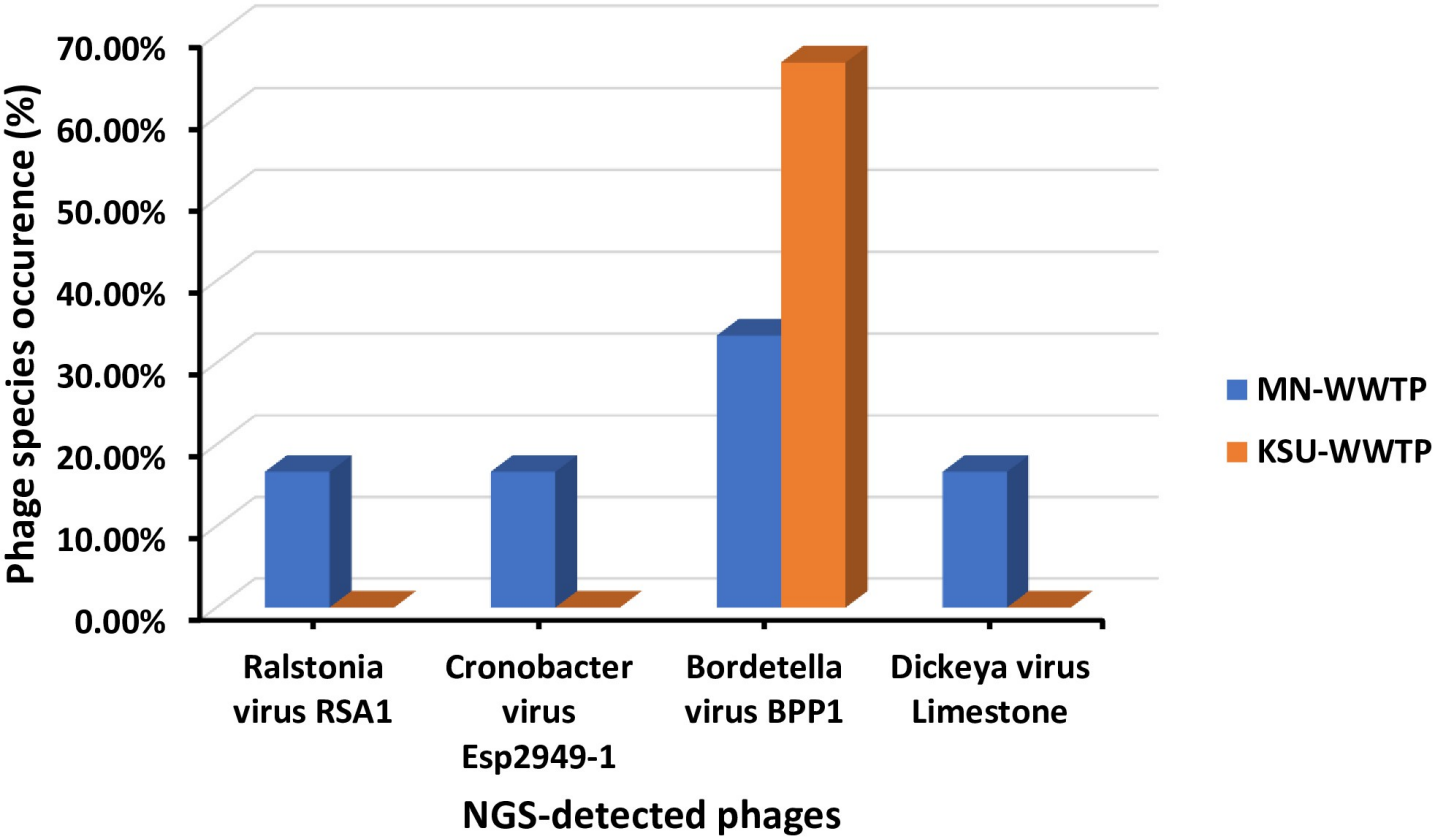
Occurrence of bacteriophages in NGS samples in WWTPs.

KSU-WWTP showed the highest occurrence of Bordetella virus BPP1 (67%, Fig 7). Bordetella virus BPP1 was found significantly correlated to KSU-WWTP influents (*p* = 0.001). On the contrary, MN-WWTP depicted higher bacteriophage occurrence of 16.6% for Ralstonia virus RSA1, Cronobacter virus Esp2949-1 and Bordetella virus BPP1 (Fig 7).

#### 3.5.3. Temporal-based relative abundance of NGS-detected phage species

**Fig 8:**
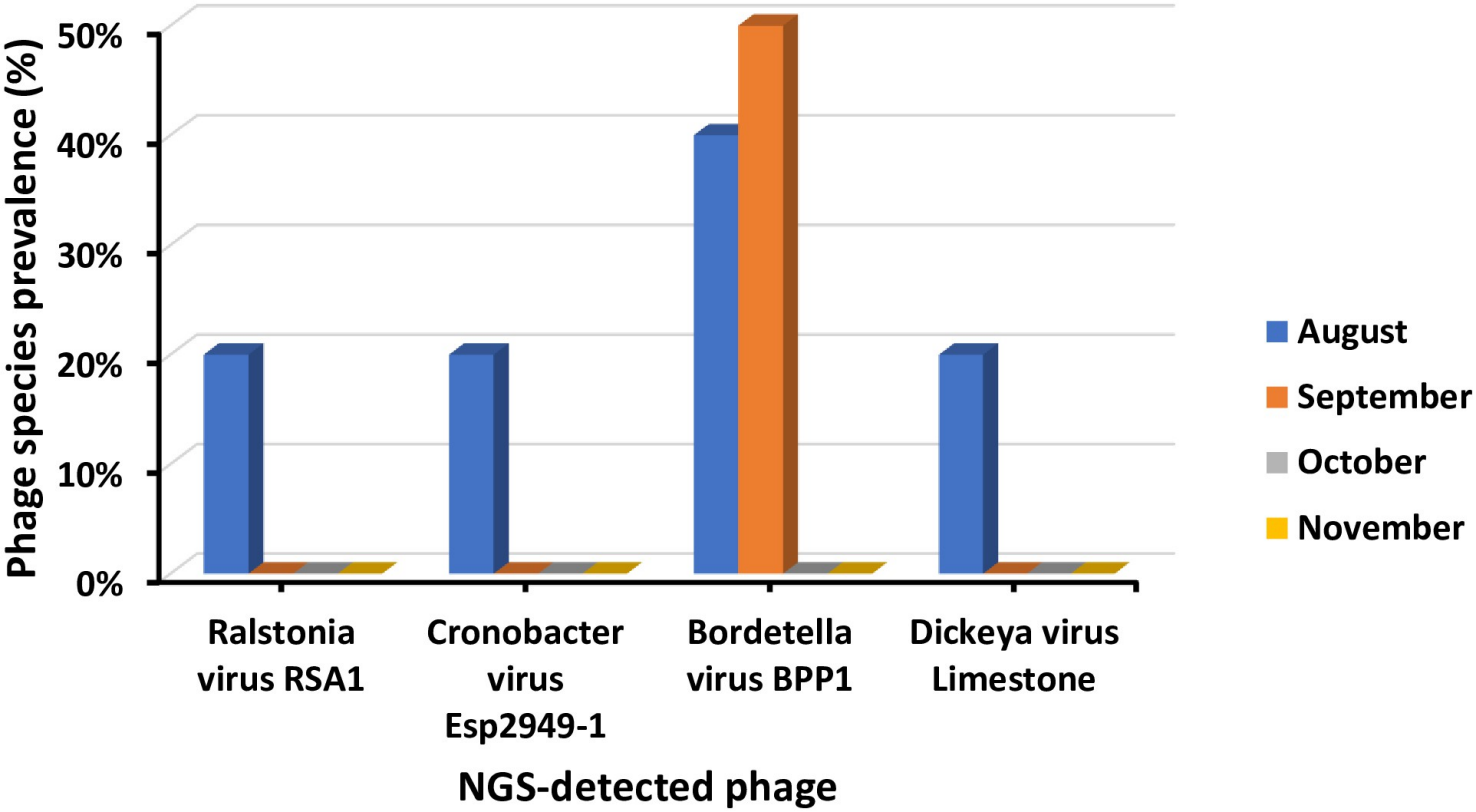
Occurrence of bacteriophages monthly in NGS samples.

Bordetella virus BPP1 was detected with the highest occurrence amongst phage species in September (50%) followed by August (40%). Moreover, all four-phage species were detected in August. Whereas Cronobacter virus Esp2949-1, Dickeya virus Limestone and Ralstonia virus RSA1 were absent from September to November (Fig 8). Furthermore, significant influence of temporal variations on phage sequence abundance was detected only in case of Bordetella virus BPP1 (*p* = 0.015).

## 4. Discussion

Phages are vital for renewal of organic matters, provisions of nutrient supply cycles, serve as genomic reservoirs and drive the bacterial diversity. Several studies has investigated the occurrence of bacteriophages in various water sources [2, 26, 27]. Moreover, bacteriophages detection has been considered as important indicator for fecal pollution [28]. Therefore, monitoring of bacteriophage in water, of particular interest, WWTPs has become a routine work for control of fecal contamination associated concerns [22]. For instance Al-jassim has detected bacteriophages from domestic water treatment plant in Jeddah, Saudi Arabia [29]. Likely, we detected several bacteriophages in MN-WWTP and KSU-WWTP in Riyadh, Saudi Arabia. Bacteriophages’ prevalence results varied according to the detected family and water site. A recent global viral abundance study showed *Microviridae* to be the highest abundance phage family across the globe, followed by *Siphoviridae*, *Myoviridae*, and *Podoviridae* [30]. Unlikely, our study found *Myoviridae* family to be the most abundant phage family that could be due to geographic variations [31]. Our study focused on limited country data in comparison to the global study [30] that excluded our study area. Korf, et al. reported the higher existence of *Myoviridae* as compared to *Siphoviridae* that agrees with our findings [32]. Whereas Jurczak found more *Siphoviridae* abundance as compared to *Myoviridae*, and reported the least *Podoviridae* abundance in coliphages from sewage [33]. The disagreement with Jurczak findings could be owing to the water sources, types and spatial differences. Phage families prevalence was influenced temporally, with highest prevalence detected in August, followed by September, which partially agrees with a previous study that characterized coliphages form River Nile and five wastewater drainage during summer and winter seasons [22]. Moreover, the latter study detected three different types of phages found related to *Myoviridae*, *Podoviridae* and *Siphoviridae* families that is in line with our findings. Furthermore, A study of viral communities of Lake Baikal indicated the dominance of different families *like Myoviridae*, *Siphoviridae* and *Podoviridae* that supports our results [34]. Other study has recorded relative abundance of 34% and 26% for *Siphoviridae* and *Myoviridae*, respectively in wastewater treatment plant samples [35]. The discrepancy of the latter study findings is owing to geographical and meteorological differences.

The phage prevalence was found affected by temperature conditions being entirely undetectable at ≤29°C. Similarly, bacteriophages abundance was reported elsewhere to be highly influenced by the environmental conditions and the presence of host cell [30, 36, 37]. For instance, the abundance level of Cyanobacteria was found increasing with temperature rise, resulting in the increase of the associated phage community that supports our results [38]. Moreover, viral communities in the latter study were found dominated by dsDNA viruses’ families like *Myovirdae* and *Siphoviridae* in agreement with our study.

In the present study, four bacteriophages were detected by NGS-Sequencing with various abundances. Cronobacter virus Esp2949-1 was detected for the first time in Saudi Arabia and was recorded with the highest abundance. Likewise, Lee, et al. has detected the same Esp2949-1 phage in sewage samples in South Korea [39]. Cronobacter phage was also identified in 12 raw wastewater samples in Yangzhou, Jiangsu, China with 10 variants showing greater diversity among phage strain level [40]. Unlikely, our findings revealed a single Cronobacter phage strain, Esp2948-1. However, population density, life style and living standards can lead to phage diversity [31]. Bacteriophages are known of their significant ability and specificity to infect defined bacteria including pathogenic bacteria in a wide range of environments. Cronobacter virus Esp2949-1 could be utilized as a potential virulent agent against neonatal and infantile pathogen *Cronobacter sakazakii*, known to cause enterocolitis, meningitis and septicemia outbreaks globally [39, 41]. Bordetella virus BPP1 was the second abundant phage in our sequenced samples. Bordetella virus BPP1 was previously detected in WWTPs in Jeddah, Saudi Arabia with the lowest abundance that disagrees with our findings [42]. In contrast, other Bordetella phages (CN1, CN2, FP1, MW2, and LK3) were isolated from surface water from Serbia, Georgia, Hungary Egypt, Switzerland and Turkey. The Bordetella phage strain variations may be attributed to difference of water sources and spatial considerations [43]. The BPP1 phage is a close relative to Bordetella bronchiseptica phage vB_BbrP_BB8 that is known for its antibacterial application against *Bordetella bronchiseptica* [44] recognized to cause respiratory infections [45]. On the other hand, Ralstonia virus RSA1 displayed the lowest sequence abundance of 1.04% in MN-WWTP influents and least sequence abundance in KSU-WWTP influents. However, Trotereau, et al. isolated Ralstonia phages from more than 50% of different environmental samples [46]. Despite the significantly low abundance of Dickeya virus Limestone and Ralstonia virus RSA1 in our samples, it indicated the probable low abundance of their hosts which are potential plant pathogens including *Dickeya solani* (causing blackleg and soft rot of potato) [47] and *Ralstonia solanacearum* (causing bacterial wilt of tomato) [48]. The high diversity of phages among different isolation times and areas can affect the isolates quality as well as quantity due to environmental pressures [13, 31, 37].

In conclusion findings of present study will help in understanding of phage composition, diversity and ecology from complex wastewater sources.

## Supporting information

S1 Fig PCR product of raw water sample*s* from MN-WWTP. (A) Lane 1 and 9: DNA ladder (100–2000 bp), Lane 2: negative control, Lane 3–8, 10–13 and 15–16: negative samples, Lane 14: 704 bp amplicon (Myoviridae, obtained by MGF primer), (B) Lane 1 and 9: DNA ladder, Lane 2: negative control, Lane 3, 5–8 and 10–16: negative samples, Lane 4: 500 bp amplicon (Myoviridae, obtained by CTF primer), (C) Lane 1 and 9: DNA ladder, Lane 2: negative control, Lane 3–8 and 10–15: negative samples, Lane 16: 459 bp amplicon (Siphoviridae, obtained by MCF-2 primer), (D) Lane 1 and 9: DNA ladder, Lane 2: negative control, Lane 3–8, 11–13 and 15–16: negative samples, Lane 10: 1278 bp amplicon (Myoviridae, obtained by MCF-1 primer) and Lane 14: 500 bp amplicon (Myoviridae, obtained by CTF primer), and (E) Lane 1: DNA ladder, Lane 2: negative control, Lane 3–6 and 8: negative samples, Lane 7: 500 bp amplicon (Myoviridae, obtained by CTF primer).
(PDF)

S2 Fig PCR product of raw water sample*s* from KSU-WWTP. (A) Lane 1 and 9: DNA ladder (100–2000 bp), Lane 2: negative control, Lane 3–7 and 10–16: negative samples, Lane 8: 459 bp amplicon (Siphoviridae, obtained by MCF-2 primer), (B) Lane 1 and 9: DNA ladder, Lane 2: negative control, Lane 3–5, 7–8 and 10–16: negative samples, Lane 6: 500 bp amplicon (*Myoviridae*, obtained by CTF primer).
(PDF)
