# Supplementary figures and images for: Novel findings in context of molecular diversity and abundance of bacteriophages in wastewater environments of Riyadh, Saudi Arabia
Source: PLoS One. 2022 Aug 18;17(8):e0273343. doi: 10.1371/journal.pone.0273343 (PMC9387821; doi:10.1371/journal.pone.0273343)

(A)

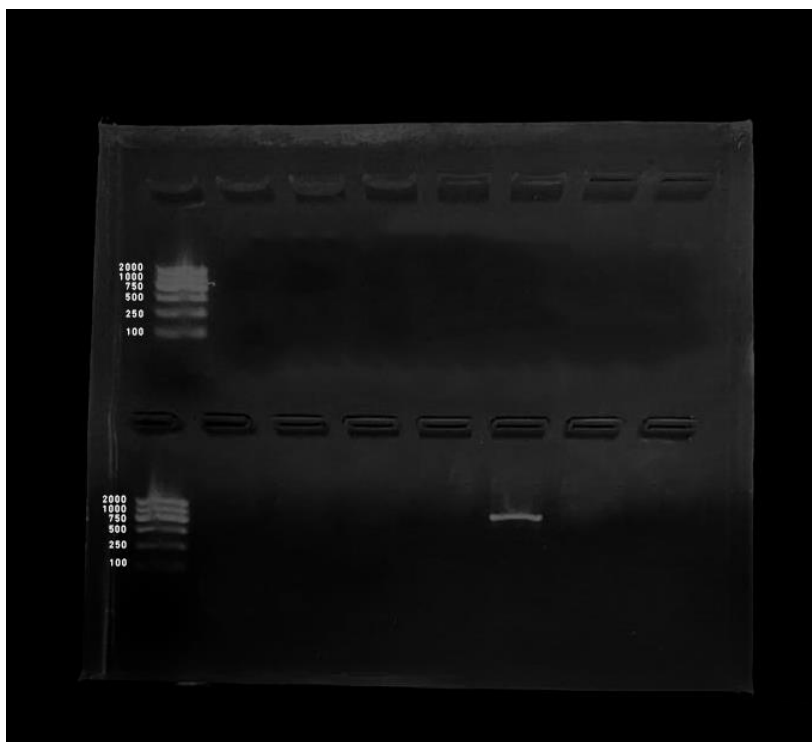

(B)

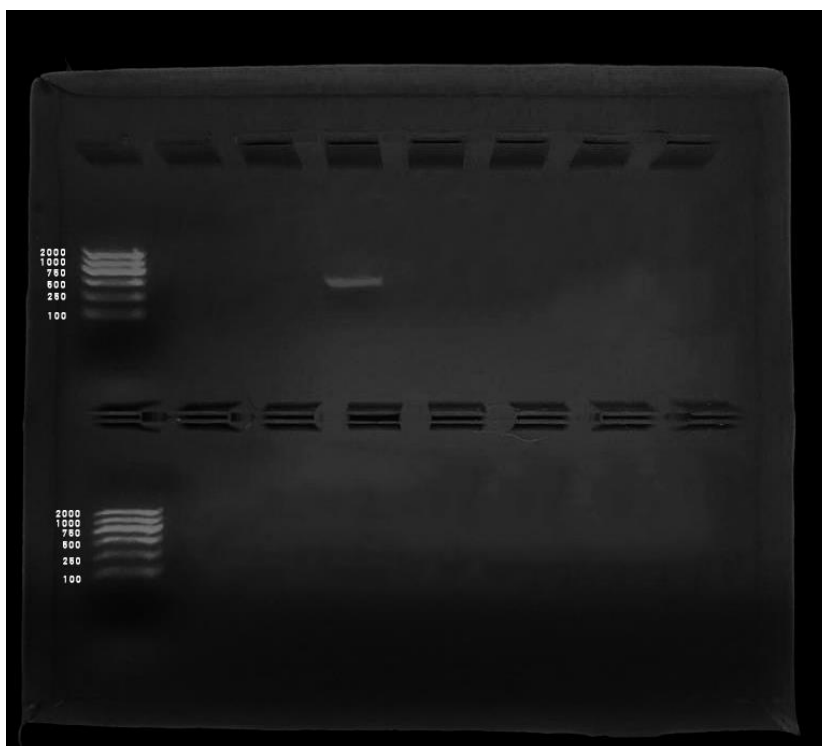

(C)

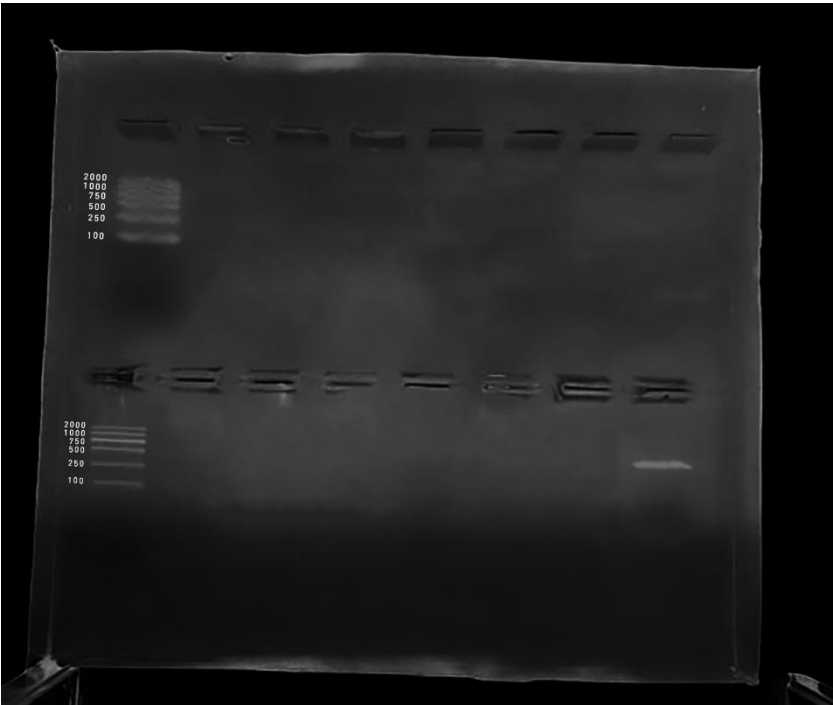

(D)

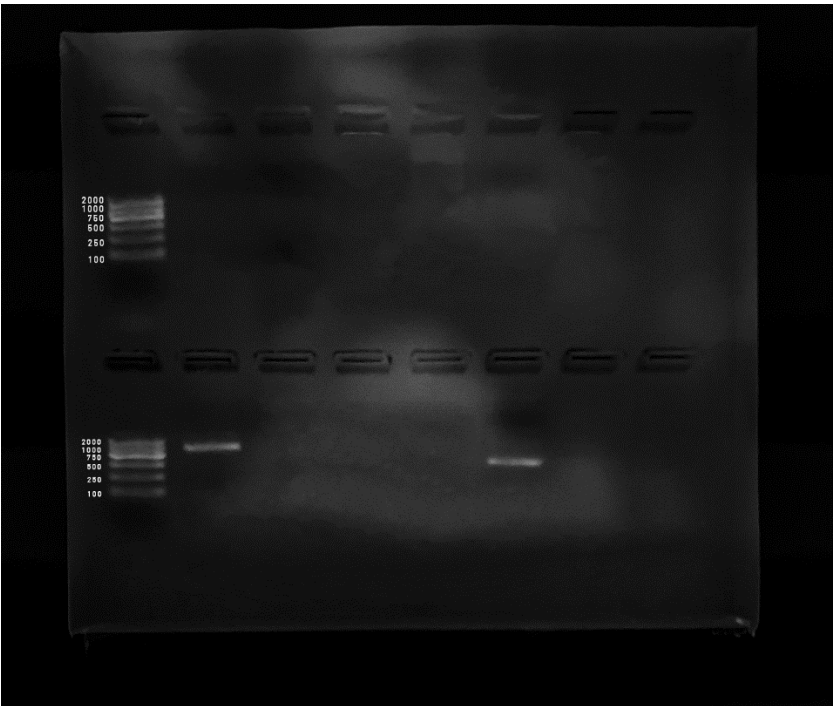

(E)

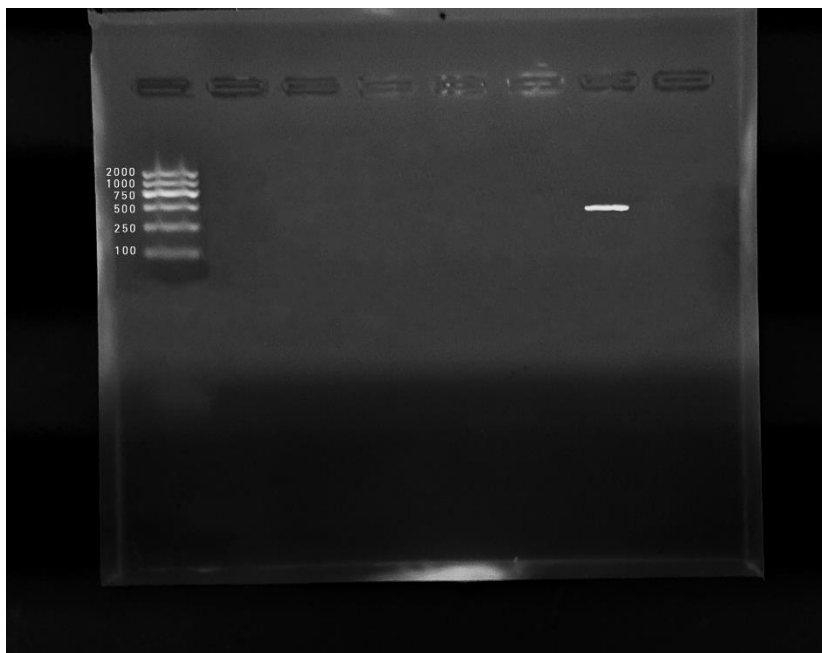

**S1 Fig.**

Supplement: S1 Fig — (A) Lane 1 and 9: DNA ladder (100–2000 bp), Lane 2: negative control, Lane 3–8, 10–13 and 15–16: negative samples, Lane 14: 704 bp amplicon (Myoviridae, obtained by MGF primer), (B) Lane 1 and 9: DNA ladder, Lane 2: negative control, Lane 3, 5–8 and 10–16: negative samples, Lane 4: 500 bp amplicon (Myoviridae, obtained by CTF primer), (C) Lane 1 and 9: DNA ladder, Lane 2: negative control, Lane 3–8 and 10–15: negative samples, Lane 16: 459 bp amplicon (Siphoviridae, obtained by MCF-2 primer), (D) Lane 1 and 9: DNA ladder, Lane 2: negative control, Lane 3–8, 11–13 and 15–16: negative samples, Lane 10: 1278 bp amplicon (Myoviridae, obtained by MCF-1 primer) and Lane 14: 500 bp amplicon (Myoviridae, obtained by CTF primer), and (E) Lane 1: DNA ladder, Lane 2: negative control, Lane 3–6 and 8: negative samples, Lane 7: 500 bp amplicon (Myoviridae, obtained by CTF primer). (PDF) [file pone.0273343.s001.pdf]

(A)

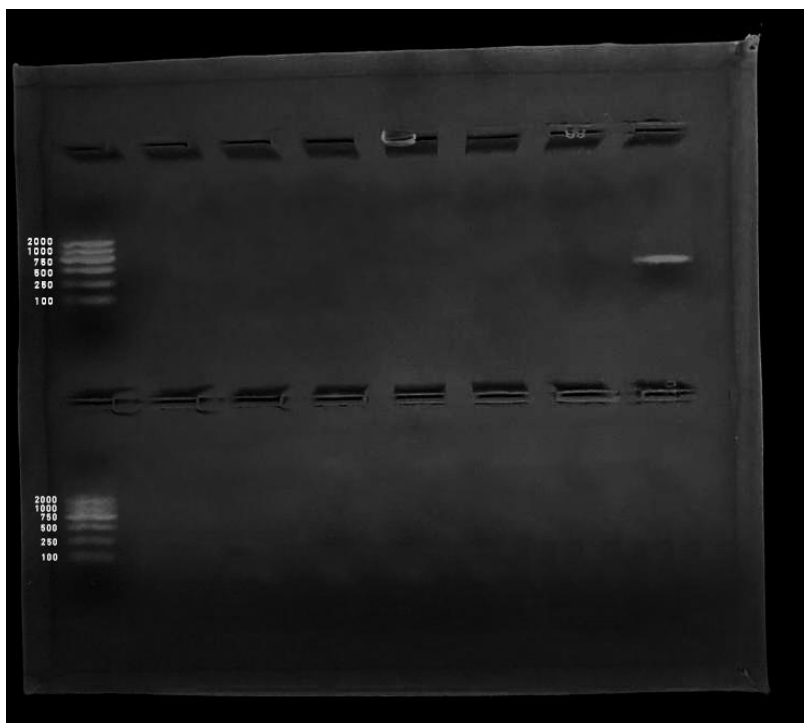

(B)

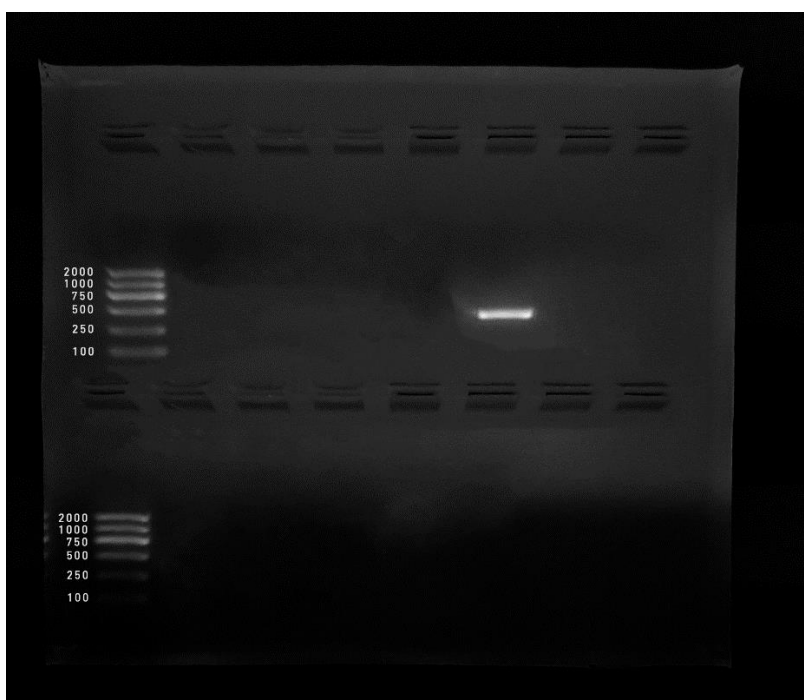

S2 Fig.

Supplement: S2 Fig — (A) Lane 1 and 9: DNA ladder (100–2000 bp), Lane 2: negative control, Lane 3–7 and 10–16: negative samples, Lane 8: 459 bp amplicon (Siphoviridae, obtained by MCF-2 primer), (B) Lane 1 and 9: DNA ladder, Lane 2: negative control, Lane 3–5, 7–8 and 10–16: negative samples, Lane 6: 500 bp amplicon (Myoviridae, obtained by CTF primer). (PDF) [file pone.0273343.s002.pdf]
